# Supplementary figures and images for: The actin binding sites of talin have both distinct and complementary roles in cell-ECM adhesion
Source: PLoS Genet. 2024 Apr 25;20(4):e1011224. doi: 10.1371/journal.pgen.1011224 (PMC11075885; doi:10.1371/journal.pgen.1011224)

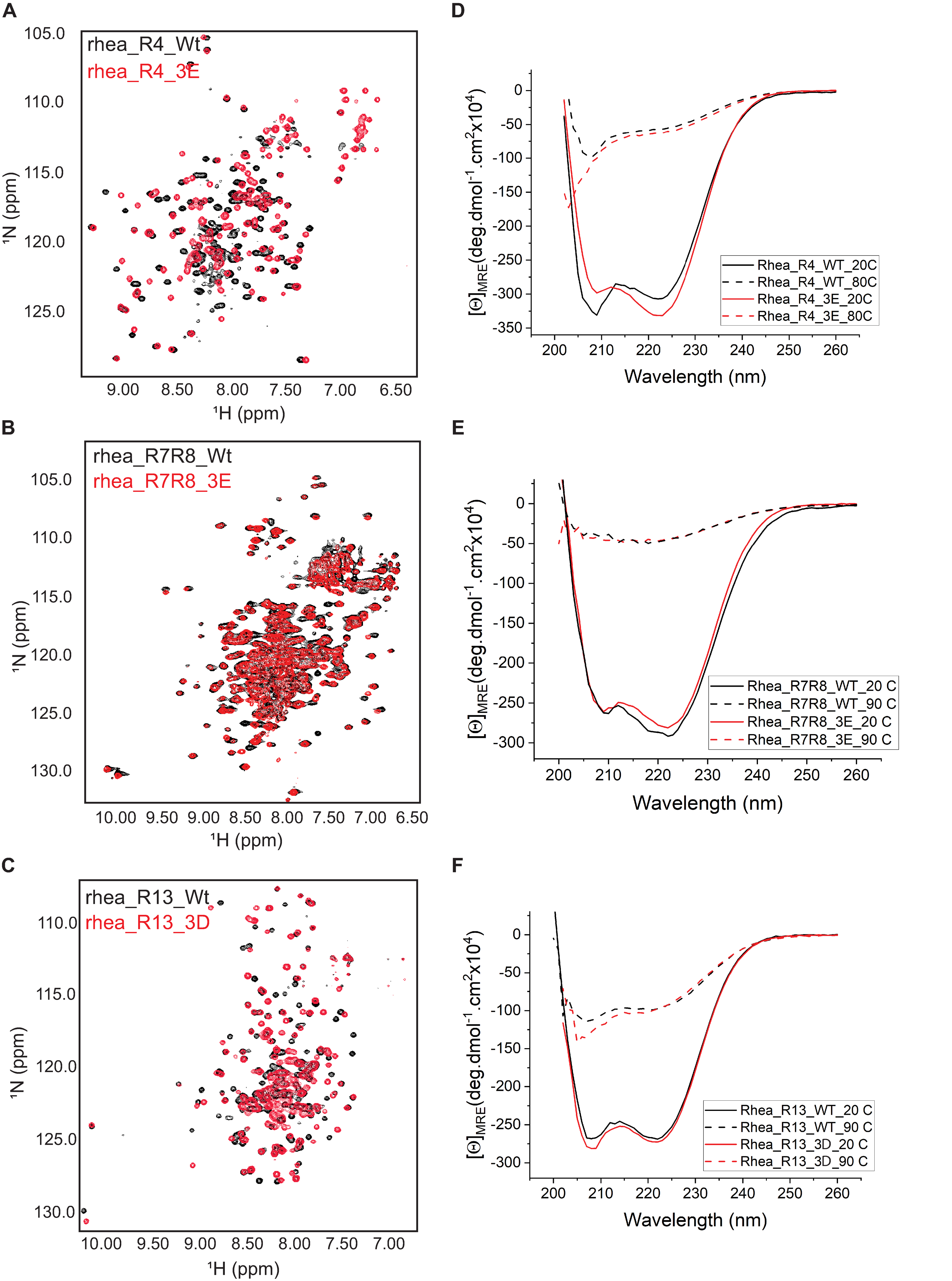

Supplement: S1 Fig — (A-C) 1H,15N HSQC spectra recorded at 25°C of the Wt (black) and mutant (red) variants of (A) R4, (B) R7-R8 and (C) R13 domains. (D-F) CD spectra of the Wt (black) and mutant (red) variants of (D) R4, (E) R7-R8 and (F) R13 domains at 20°C and 90°C. (TIF) [file pgen.1011224.s001.tif]
