# Supplementary material for: The actin binding sites of talin have both distinct and complementary roles in cell-ECM adhesion
Source: PLoS Genet. 2024 Apr 25;20(4):e1011224. doi: 10.1371/journal.pgen.1011224 (PMC11075885; doi:10.1371/journal.pgen.1011224)
Supplement: Attachment — Submitted filename: ABS response to reviewers.docx [file pgen.1011224.s003.docx]

**Dear Pablo and Gregory,**

**Enclosed for your consideration please find our response to the reviews of our manuscript entitled “The actin binding sites of talin have both distinct and complementary roles.”. We were happy to see that the reviewers found our work to be of interest and were grateful for the helpful and constructive suggestions we were provided with.**

**The reviewers raised important points that required us to obtain additional data, modify our data presentation in several instances, as well as to change and enhance the manuscript. In line with the reviewer feedback we added 2 new major experiments and significantly changed the text adding caveats, providing more precise explanations, and improving the way the data was presented.**

**Some of the key improvements to the manuscript include:**

1) **A new experiment imaging larval musculature to better understand why our mutants die during those stages.**
2) **A new experiment quantifying native Vinculin recruitment in our mutants as opposed to relying only in overexpressed Vinclin as we did previously.**
3) **Adding a comprehensive schematic model to better explain how we think the two different actin binding domains in Talin works**
4) **Clarify the experiments using the K17E allele to illustrate that ABS3 can specifically recruit ABS2 to the membrane**

**A detailed reply to the reviewers’ concerns and criticisms is appended below. We believe that this new version of the manuscript is substantially improved and enhanced, making our paper more impactful and complete. We hope you find that the work is now suitable for publication.**

**Best Regards,**

**Guy Tanentzapf + Darius Camp**

**Point by point response to reviewer comments:

Reviewer #1:**

1) This paper will tremendously benefit of showing a schematic model diagram of how the authors think the two different actin binding domains in Talin works at the end of the paper (e.g. analogous to a graphical abstract). While the authors do a good job explaining how the genetic results should be interpreted in text, it would be great if they can provide a visual representation of a Talin (or Talins) working as a dimer and how the two domains are providing different functions, especially for researchers who are not in the Talin/Integrin/Actin field.

**> We agree that a model would greatly improve the paper and help the reader understand our model based on genetic interactions that are sometimes hard to grasp. We have therefore included a comprehensive diagram at the end of our paper Fig 8 K summarizing our findings.**

2) Line 93 and elsewhere: The authors sometimes italicize the word ‘Drosophila’ and sometimes they do not. They should be consistent (I personally recommend italicizing it everywhere).

3) Line 102: When the author say “flies have only one talin gene”, they should mention that this gene is called ‘rhea’ here. Otherwise, some readers may fail to make the connection between talin and rhea later in the paper.

4) Line 166 and line 169: URL for FlyCRISPR website does not have to be shown in the main text. It would be sufficient to refer to it in the Methods section, and you can just site the relevant manuscript here, which I believe is Ref 47.

5) Lines 227-227 and 232: ‘Retraction’, ‘Dorsal’ and ‘Closure’ do not have to be capitalized.

6) Line 260: ‘Cell’ doesn’t have to be capitalized here.

7) Line 459: ‘was’ should be ‘were’.

8) There may be several typos regarding amino acid numbers throughout the text.

Please check carefully when finalizing the work.

line 136, 144: 940(correct residue should be: 941), 941(942), 948(949)

line 140, 145, 159, 180: 2670(2450), 2671(2451), 2672(2452)

line 157, 176: 940D(941E), 941D(942E), 948D(949E)

**> Thank you for carefully reading our paper and noticing these mistakes. They have all been corrected.**

**Reviewer #2:**

1) One key aspect for the conclusions is that the binding capacity of the ABS is truly knocked out with the chosen mutations, especially for ABS3 since the weaker phenotypes of this mutant leads the arguments towards the unique versus collaborative work of the two ABS, so is the actin binding capacity of the ABS3 mutants truly gone in vivo? I think the authors should check this, maybe by ipps from embryos?

**> We agree that information about actin binding capacity of the mutant versions of talin is an important bit of information and we should have been clearer about this issue. There are four things we would like to emphasize in this regard: first, we used the strongest mutations known in both ABS2 and ABS3 that specifically abolish actin binding and are not thought to impact other functions of Talin. Second, although these mutations likely reduce actin binding significantly, we would not assume that they completely abolish actin binding. Third, in our interpretation of the data we took into account the possibility that the talin protein containing the mutations in ABS2 and ABS3 that we used still has residual actin binding activity. Fourth, our previous genetic analysis of a deletion mutant (the *rhea*^15-39^ allele; France-Cea et al 2010) that lacks the entirety of the ABS3 domain shows that it gives rise to phenotypes that are comparable to those we describe in our KVK/DDD ABS3 mutant. In line with the first point, we state in the 1st paragraph of our result section that the ABS2 mutations we used “reduced the actin binding ability of talin, as judged by *in vitro* actin sedimentation assays, by over 60%** [**(Kumar et al)**](https://www.zotero.org/google-docs/?fIDi0O)**” while the ABS3 mutations we used “greatly reduce actin binding through ABS3”. Consequently, cautious interpretation of the data should consider both ABS2 and ABS3 mutants as hypomorphic alleles that still have some capacity to bind actin. Previous analysis of the KVK/DDD ABS3 mutants (Gingras et al, 2008; Franco-Cea et al., 2010) found anywhere between 40%-73% decrease in actin binding in an *in vitro* actin co-sedimentation assay. These *in vitro* experiments likely represent an overestimate of the actin binding activity of these mutations as they are designed to optimize actin binding. Nonetheless, we agree with the reviewer that this is important context for our readers to keep in mind and we modified the text to directly state this (middle of the results first paragraph, lines 168-175 in track change document). As the reviewer points out the probable hypomorphic nature of the ABS mutants is less of an issue with ABS2 as they have such a strong phenotype but the question arises as to whether an absolute loss of ABS3 mediated actin binding would give rise to a stronger phenotype. This question is answered by the *rhea*^15-39^ allele (France-Cea et al 2010) that completely deletes ABS3 but does not give rise to a significantly stronger phenotype than the KVK/DDD allele. Moreover, the vertebrate equivalent of the R2531G allele that we use in this paper is thought to abolish actin binding in addition to dimerization yet, in terms of adhesion phenotypes, does not give rise to a significantly stronger phenotype than the KVK/DDD allele (Gingras et al, 2008; Franco-Cea et al., 2010). Even so, we tried our best to be very cautious in interpreting the phenotype of the ABS3 mutants and in fact took advantage of its hypomorphic nature for powerful genetic interaction studies with Vinculin, and ABS2 mutants. We believe all of this is important context and we inserted text to include this information and added substantially to the discussion to specifically address this issue in (discussion last paragraph).**

2) The authors say: ‘Using the approach detailed here we successfully generated multiple independent mutated lines of ABS2 and ABS3’…how many lines have been tested for the phenotypes described all long the MS? Is it just one line of each for all experiments?

**We agree that this statement needs clarification as different lines could potentially have small genetic variations leading to a range in phenotypes. Once the lines were established we ensured they behaved, generally speaking similarly, and then proceeded to focus on one line in particular for each mutation. Towards clarifying this we added the following statement: “Using *the approach detailed here we successfully generated multiple independent mutated lines of ABS2 and ABS3. As the different ABS2 and ABS3 mutant lines displayed identical sequencing results, phenotypic analysis was then performed on only 1 line per ABS mutation to investigate their role*”.**

3) For wings, DC and GBR, both ABS2 and 3 mutant phenotypes are similar to the null phenotype. For muscle attachment, then ABS2 is more important. This is again an important finding for the ‘unique versus collaborative’ hypothesis. But how are muscle attachments quantified? I couldn’t find it anywhere. It says muscles round up, but in some mutant embryos is difficult to see which one is attached or not (fig3c or d for example). How was the attachment of muscles phenotype quantified?

**> We agree that the muscle detachment analysis needs to be better explained, consequently we have added an extra section: “Muscle detachment analysis” in our M&M explaining our protocol.**

4) Part of the conclusion is that ABS3 is required ‘to maintain and strengthen’ integrins adhesion. How did the authors conclude this? Which experiments show this? I fail to conclude that. Yes, the phenotype is weaker than ABS2 and various proteins are clearly recruited in this mutant background, but that is not concluding that is required for maintaining or strengthening the complexes?

**> We may have overreached with that particular phrasing (which is used only once in the paper). The “maintenance” part comes from the fact that junctions are established (as evidenced by the recruitment of various adhesion components) but we do see an adhesion phenotype in terms of a degree of muscle detachment, wing blisters and morphogenetic defects). If the adhesions form and adhesion complex components are recruited and yet an adhesion phenotype is observed this suggests a defect where the adhesions are either not strong enough, too unstable, or both too weak and too unstable. But we agree this is perhaps too speculative. We therefore changed the wording in that specific section to say “*In comparison, ABS3 mutants are recruited to sites of adhesion where they assemble is required to maintain and strengthen adhesions, but exhibit a variety of adhesion defects*“**

5) Related to this, the rap1-binding mutant rheaK17E is a nice experiment and shows that ABS3 mutant proteins can recruit ABS2 mutant proteins when together in the cells. For strengthening this experiment, the authors should check rheaK17E levels of expression.

**> This is an excellent point, and we have indeed studied K17E expression levels in detail in our previous paper dedicated to this mutant (Camp et al. 2018). Specifically we used western blots, qPCR and immunostainings to show that K17E, similarly to ABS2, is expressed at levels that are comparable to those observed for wildtype talin but is not recruited to MTJs. We now mention this in the text (lines 441-447).**

6) The authors say: ‘To test if these differences were due to differences in the expression of the ABS mutations, we carried out qRTPCR experiments which showed that both the ABS2 and ABS3 mutant…’ The authors should also check protein levels by western blot, as translation or stability of the protein could be different.

**> This is a very good point as precisely measuring protein levels by western blot would demonstrate that ABS mutant proteins are made and stable. This information is key to interpreting the structure function data presented here and we have therefore rewritten the talin recruitment section (ABS2 but not ABS3 is required for talin recruitment to sites of adhesion) to make it more straightforward.**

**Due to its large size (270Kd) detecting Talin levels by western blots is known to be technically challenging and despite our best efforts we were unable to reliably obtain quantifiable Talin Western Blot data for all our mutants.**

**However, an alternative way to quantify Talin expression is using immunohistochemistry to study Talin recruitment to the sites of adhesion. Our analysis of Talin recruitment data (FIG 4K) supports the conclusion that both the ABS2 and ABS3 mutant proteins are translated and stable:**

**In (FIG4 F & H) we show that Talin ABS3 mutant protein is enriched at sites of integrin-mediated adhesion at MTJs at similar levels to those seen in the WT showing that the protein is made, stable and properly recruited.**

**Unlike the homozygous ABS3 mutant, the homozygous ABS2 mutant protein was not detected at sites of adhesion at MTJs in mutant embryos (FIG4 D & E, quantified in Fig. 4K). We were able to overcome this issue using a different approach to allow us to visualize the expression of the ABS2 protein. In particular, we took advantage of our observation that the Talin null mutants heterozygotes (*rhea/+*) have about half as much talin in sites of adhesion at muscle attachments as the homozygous wildtype (+/+). In comparison in rheaABS2/+ heterozygous embryos have about 80% the levels of Talin recruitment to sites of adhesion (FIG4 C,G, quantified in Fig. 4K). This confirms the ability of a substantial population of the ABS2 mutant protein to be translated and recruited to the membrane. While this does not imply that the ABS2 mutation has no impact on stability and translation, it suggests that if such an impact exists it is unlikely to give rise to the strong, null-like, phenotypes we see in ABS2 mutants.**

7) Why is the talin transgene not rescuing adult viability (fig2A)? This may be well known in the field, but it should be explained here too.

**> We unfortunately do not have a good answer for this. It could be that in the transition to adulthood some cell populations do not express the Ubiquitin-E63 as well as they do during embryonic or pupal stages. What we do know for sure is that we have used this rescue line multiple times over the years (Tanentzapf et al NCB, 2006; Franco-cea et al, 2010; Ellis et al, 2011, 2013, 2014) and we cannot detect a difference between wildtype and wildtype rescue during embryonic or larval stages. We now specifically mention this in the materials and methods section: “Fly stocks and genetics”**.

8) Can MHC levels also be quantified, as the authors did for F-actin? This measurement would contribute to understanding not just actin recruitment, but contractility capacity at the complex.

**> We agree that measuring MHC levels would provide valuable insight on muscle function and help us better characterize each mutant. Unfortunately, technically we don’t have a way to do this reliably. The reason for that is that unlike measuring components specifically recruited at the MTJ (like actin accumulation), analyzing muscle fiber components, that concentrate throughout the cytoplasm, is unreliable in mutants due to the wide range of muscle morphology they display. As shown in figure 4 the untethered muscles in talin null or ABS2 mutants severely contract, meaning they concentrate muscle fiber proteins in a smaller area. This causes an artificial increase in the intensity of the MHC staining as all it concentrates in this smaller region. Meanwhile, undetached muscles retain their stretched morphology and consequently MHC appears less bright as it is distributed over a larger area.**

9) When studying the recruitment of complex components (fig6), how do the authors know where the MTJs are if they are not labelled with a 3rd component that is not affected in the mutant background? As in previous images, a marker such as integrins (can integrins be used?) should be used in combination with paxillin and pinch, so the MTJs can be identified and then the level of recruitment to those sites of paxillin and pinch can be measured.

**> This is an excellent question and is the result of us not being clearer in our description of how these experiments were carried out. We failed to explain that we always use alphaPS2 integrin staining in another channel when we image in order to identify where the MTJs are located. This is made possible by integrin recruitment being independent of Talin (Brown et al, 2002). To make this clearer we modified figure 6 to by adding the integrin channel, labeled in a different color, to the images to show the localisation of integrin, we also altered the figure legend accordingly. We were previously reluctant to add another channel to the images because we worried it would make the staining of the measured protein less visible and the figures would be less intuitive. However the reviewer comment convinced us that this would be a valuable addition. We opted against the third option of showing all the channels individually as this would double the numbers of the panels in this figure making it very hard for the reader to understand.**

10) Contrary to what the authors claim, I think vinculin has a substantial effect on viability, when looking at L1 survival rate (SR): compared wildtype larvae in fig2b (100% SR) with wildtype + vinculin in fig7B (68.2% SR), Similarly, larvae motility phenotype in ABS3/3 and ABS2/3 mutant is nicely rescued by vinculin, but I fail to see this for L1 SR, which is lower in fig7 than in fig2. This section needs to be more precise and accurate. This is important, as it may suggest that the role of vinculin-talin interaction may be different in different contexts.

**> The reviewer raises an interesting point which we missed. Unfortunately, we took Maartens et al’s (2016) conclusion that “Expression of wild-type, full-length vinculin (UAS vinc-FL) either in the muscles (with *Mef2::Gal4*) or ubiquitously (with *Act5C::Gal4*) did not affect viability” uncritically. In fact, when we went back to take a closer look at Maartens et al’s own data, overexpression in muscles of the wildtype Vinculin gave about a 10% decrease in viability compared to control. Our data indeed shows a 30% decrease in viability and loss of motility across all genotypes we tested. In line with the reviewer’s request that we be “*be more precise and accurate*” we rewrote this section to make all of this explicit. Our main result, which is not impacted by these changes, is that despite causing some overall negative impacts on viability, vinculin overexpression induces rescue of mobility phenotypes and allows survival of some ABS3 mutants well passed where they usually die without vinculin overexpression.**

11) In the vinculin-RFP experiment the flies have also endogenous vinculin, right? In this case, this experiment is looking at vinculin recruitment in conditions of vinculin overexpression. I think the authors need to do the same experiment without OE of vinculin, by antibody staining of endogenous one, or by an endogenous vinculin tagged.

**> We agree! There is no reliable Vinculin antibody in flies but there is an excellent genomically tagged GFP construct (**[***Pinheiro et al., 2017***](https://www.nature.com/articles/nature22041)**) which we obtained and used to repeat the experiment. We now include this analysis in panel modified figure in panel O in the updated figure 7 (and described in lines 662-688). Overall, we get similar results, vinculin recruitment is very low or absent in null or ABS2 talin mutants but is fairly robust (if not quite wildtype) in ABS3 and ABS2/ABS3 transheterozyogte mutants.**

12) Not an expert in the topic, but there are various relevant papers published in 2022 and 2023 that are not mentioned in this MS. (e.g., The C-terminal actin-binding domain of talin forms an asymmetric catch bond with F-actin, PNAS 2022. Owen et al.). The reference list needs updating, and more importantly, the findings need to be presented in the light of those papers, especially in the Discussion.

**> Thanks for reminding us about the paper from Owen et al which is now mentioned in the discussion (lines 842-847).**

13) Minor point: the pinch antibody description is duplicated, but with different information, PINCH (rabbit, 1:1000; Mary Beckerle, University of Utah) or Mary Beckerle, Huntsman Cancer Institute, UT)

**> Thank you for bringing this to our attention, this error was fixed.**

**Reviewer #3:**

1. It would be interesting to better understand, why the ABS3 mutants die as L2 larvae, despite having only weak phenotypes in the embryo. The authors suggest that ABS3 is need to maintain the integrin adhesion complex at the MTJ. This could be easily tested in L2 larval filets stained with phalloidin. The high sarcomere forces produced at that stage may result in more obvious detachments.

2. Similarly, the ABS2/ABS3 transhets could be investigated at L3 stage to quantify possible muscle detachments. This would provide evidence that the proposed Talin mutant dimer is functionally limited to a low force regime.

Do these mutants make mature pupae that fail to eclose or do they die at early pupal stages, which would rather indicate a larval problem?

**> We agreed with the reviewer that we should analyze muscles and MTJs in ABS3 mutants and in ABS2/ABS3 transheterozygotes. Consequently, we introduced the muscle marker actinin::GFP into the background of our mutants in order to visualize muscle structures during larval stages. As shown in the new panels I-L in figure 2 (and discussed on lines 268-280), we looked at the mutants in the latest larval stage that they were able to reach, L2 for ABS3 mutants and L3 for ABS2/ABS3 transhet. Since null allele of talin and ABS2 mutants die during early L1, they could not be imaged with this approach as they proved too fragile for the staining protocol. Surprisingly, compared to wildtype we were not able to find noticeable muscle defects in ABS3 mutants or ABS2/ABS3 transheterozygotes indicating that lethality occurs before visible muscle defects develop. This is consistent with our other observation that some transheterozyotes escapers can reach pupal stages during which they die. Death occurs throughout the pupal stages in the transheterozygotes making hard for us to draw conclusions. Unfortunately, these findings did not provide us with clear answers and we still do not know why ABS3 and the transheterozygotes die, talin is expressed ubiquitously in fly larva and, in theory, can play a role in a a great number of essential homeostatic processes: digestion, neural function, circulation, maintenance of epithelial integrity. Unraveling this mystery will be the subject of future work in our lab.**

Minor:

1. As the manuscript talks about 3 actin binding sites in the introduction, it might be useful to include the N-terminal ABS1 in Fig. 1A as well.

**> This is a very good point, we added ABS1 to Fig. 1A.**

2. I was not able to follow the rational of the TalinK17E mutation combined with the ABS2 mutant. ABS2 homozygous is not recruited to the MTJ, null is obviously not recruited. ABSS2/null was not tested but likely will not be recruited. Is K17E homozygous recruited? If not, I doubt we learn much from this experiment and I suggest to delete it for clarity (or explain it better).

**> We agree that we should have done a better job explaining this experiment and we rewrote this section accordingly (lines 441-542). As we now explain, we sought to test the hypothesis that a main causative defect in ABS2 mutants was their failure to localize to, or be retained at, the membrane, and that this defect could be overcome by synergistic interactions with ABS3 mutants. To this end we used an allele of talin, *rhea^K17E^*, that contains a single point mutation that interferes with the direct binding of talin to Rap1, an interaction that we showed previously does not impact the expression of talin but disrupts the proper recruitment and/or retention of talin to the membrane** [**(Camp et al 2018). C**](https://www.zotero.org/google-docs/?GJ92fu)**onsequently, homozygous *rhea^K17E^* mutants behave like talin null mutants, even though the talin protein is made in wildtype amounts. Our hypothesis predicted that, unlike ABS3 mutants, *rhea^K17E^* and *rhea^ABS2^* mutants share a molecular defect in terms of recruitment. Therefore, while ABS3 and ABS2 mutants act synergistically, ABS2 and K17E mutants do not. This makes sense when taken together with our data supporting Talin dimerization. In an ABS2/ABS3 talin dimer the ABS3 mutant protein help recruit ABS2 mutant protein to the membrane where it can function. However, in an ABS2/K17E dimer neither talin is recruited and the result is no Talin at the membrane and a null like phenotype.**
